# A qualitative exploration of the dynamics of guilt experience in family cancer caregivers

**DOI:** 10.1007/s00520-023-08060-3

**Published:** 2023-10-27

**Authors:** Nur Atikah Mohamed Hussin, Nursahira Sahiba Mohd Sabri

**Affiliations:** 1 Faculty of Social Sciences (Health), Tampere University, Tampere, Finland; 2 Universiti Sains Malaysia, George Town, Malaysia

**Keywords:** Guilt, Cancer, Family caregivers, Qualitative

## Abstract

Caregiving for cancer patients can cause stress among family caregivers. Caregiving stress is also associated with guilt as they cannot provide adequate care for cancer patients. However, guilt among family caregivers of cancer patients is poorly understood. This qualitative study aimed to explore the dynamics of guilt feelings in families that care for cancer patients. This study involved 45 family caregivers of cancer patients. Thematic data analysis was conducted. There were six themes emerging. Caregiving can be challenging, guilt and blame, guilt due to lack of self-capacity, guilt for neglecting others, no guilt at all, and discussion of guilt and blame as a caregiver. This study offers insight into social workers regarding the challenges and experiences faced by family caregivers of cancer patients. Appropriate health interventions and support should be provided to family caregivers of cancer patients to improve their well-being.

## Introduction

Family caregivers of cancer patients may face challenges in their mental or physical health, social life, and financial situation as a result of caring for the patient. This is because some family caregivers may feel overwhelmed and extremely burned out owing to a lack of assistance in preparing them as family caregivers of cancer patients [45]. Furthermore, a lack of information, training, and guidance has been identified as a barrier to performing various tasks such as providing care, treatment planning, and treatment decision-making. This results in a lack of self-efficacy among family caregivers of cancer patients [12, 30, 39].

Family caregivers who provide care for cancer patients reported not being fully informed or warned about cancer recipients dying, which caused them to be fearful of losing a loved one [34]. Hospice transitions make family caregivers of cancer patients feel fear and unpreparedness, which leads to ambivalence and guilt [29]. Even though research has shown that services such as daycare, palliative care, and hospice benefit families who provide care for cancer patients [25, 41, 46], in some cultures placing cancer patients in such facilities is a disgrace [23].

Therefore, family caregivers of patients with cancer who do not obtain sufficient time away from caregiving responsibilities feel a much greater sense of burden  [35, 38]. Family caregivers of patients with cancer burden occur when they perceive their emotional, physical, social, and financial status as suffering as a result of caring for their family member [2]. Family caregivers of cancer patients may have less success managing their own physical and emotional health which can lead to high levels of stress and frustration [35]. Additionally, family caregivers of cancer patients may experience guilt if they believe they could do a better job of caring [37]. Guilt is a feeling of responsibility or remorse for falling short of obligation or expectations [35]. In the caregiving situation, family caregivers of cancer patients believe they have a responsibility to fulfil cancer patients’ expectations. As a result, family caregivers of cancer patients may experience stress and depressive symptoms [15], social withdrawal, and a continual cycle of guilt [8]. Cancer patients’ families may feel guilty [20].

However, in some situations, family caregivers of cancer patients may also experience a lack to no feeling of guilt. In fact, family caregivers of cancer patients reported experiencing positive growth, personal confidence, and self-efficacy because of their caring experiences [14, 27, 44]. Moreover, caregiving provides individuals with personal pleasure, success, and enhanced closeness in their relationship [18, 21, 32]. Furthermore, stress and guilt are not just necessarily the result of providing care to cancer patients,they are also affected by the positive appraisals and effective coping strategies of the individual [10].

Despite the fact that family caregivers of patients with cancer provide significant care and support, guilt is rarely discussed among them, especially within specific cultures [35]. This study aimed to explore the dynamics of guilt feelings in family caregivers of cancer patients and how guilt influences their caregiving experience. This knowledge is vital in providing more perspectives on cancer caregiving and improving existing caregiving quality. This study is relevant to professionals who work with cancer patients and their families.

### Guilt in family caregivers of patients with cancer

Family caregivers of patients with cancer guilt are described as a sense of inadequacy in one’s perspective in regard to the care provided to the care recipient [43]. This feeling is often negative psychologically attributed to family caregivers themselves. According to the Cancer Family Caregiving Experience Model [10], family caregivers of patients with cancer stress processes, contextual factors, and cancer progression are all critical aspects influencing caregiving families’ well-being, including guilt.

Guilt has been proposed as a major factor in the development and maintenance of depression and anxiety, implying a strong link between guilt feelings and distress [24, 35]. Guilt has been explicitly related to poor psychological and physical health in this population, as well as their self-perceptions of their capacity to cope with challenging situations [35]. Family caregivers of patients with cancer may fail to see their own condition as difficult or add guilt to their personal challenges despite not being the one with cancer [16]. Relatives also pressure themselves to be strong and prioritize the patient’s demands above their own [19, 30]. Even though family caregivers of cancer patients are aware of their own health concerns, they reported feeling guilty for not wanting to leave the patient alone [1].

Family caregivers of cancer patients require adaptation in many areas of their lives, including their professional, social, and familial lives [45]. Their roles may constrain their schedules and spontaneous plans. Despite feeling like they live in a state of suspension, family caregivers of patients with cancer often feel guilt when having fun since they feel selfish for taking time away from patients [21, 42]. On top of that guilt is added a sense of inadequacy, described as not being capable of accomplishing enough or being insufficient in the ability to fulfil the needs of the entire family while caring for one person [5, 9, 39]. Treatment costs may also affect the employment and income of family caregivers of cancer patients [3, 11, 17]. At the same time, this makes the care recipient feel guilty for bearing the burden of suffering due to cancer illness [30].

Gender has ramifications for societal expectations, social standards, and differences in guilt experience. As in many Asian societies, women have traditionally been responsible for family care, while males are expected to provide financial support for the family [22]. Caregiving is a more fundamental element of women’s character than men’s [7, 13], and female caregiving families are more involved than male caregiving families in the roles of supporting and nurturing family members (Chao & Roth, 2000,[24],Pharr et al., 2014). Women are more prone to suffering guilt [24] because they hold more responsibility for caring for all family members, resulting in a higher level of burden [4, 28, 31]. A Chinese study found that those who strongly support the “masculinity strength” gender-role standard have a more profound sense of responsibility for all caregiving tasks. This is because Chinese families value reciprocity and asking for help from a spouse may be perceived as taking advantage of the marital relationship. This makes family caregivers of cancer patients more likely to feel guilty [43].

## Methods

### Study design

This phenomenological study involved family caregivers of cancer patients. A phenomenological study seeks to understand the perceptions, perspectives, and understandings of individuals about a particular phenomenon [6].

### Participants

There were 45 participants participated in this study. The participants were family caregivers of patients with cancer (female = 30 and male = 15, ranging from 25 to 65 years of age). Relationships between family caregivers of patients with cancer and cancer patients include children, spouses, and parents. The duration of caregiving is between 3 months and more than 5 years.

### Procedure

This qualitative study obtained ethical approval from the Human Research Ethics Committee of Universiti Sains Malaysia (JEPeM) USM/JEPeM/18100450. An advertisement for this study was posted on the Facebook wall of the Malaysian Cancer Support group. In the advertisement, information about the research study, the objectives, the voluntary nature of the study, the risks involved, and the participants’ right to withdraw from the study was shared. Purposive sampling was utilized to identify the participants in this study. In order to qualify for participation, a set of criteria was established, including Malaysians over 18 years of age, family caregivers of cancer patients who provide a volunteer service every day, and those who had provided care for at least 3 months prior to the interview. This criterion is imperative to ensure rich data collection. Prospective participants were asked to share their contact information via private message. The potential was contacted. Initially, 47 potential participants were contacted, but two were excluded because of the caregiving period that is not more than 3 months. During the initial contact, the researcher explained to the participants the goals of the study, its purpose as an academic study, and their rights as participants. Mutual agreement on the date and time of the interview were decided. Participants were asked to reflect on the feelings of guilt that they experienced in their day-to-day caregiving experience, as well as the experience of caregiving. The interviews were conducted in Bahasa Malaysia. The interviews were audio-recorded. Consent was sought for audio recording. The interviews were conducted by NAMH, which has 8 years of experience conducting qualitative studies on chronic illness, bereavement, and spirituality. The demographics of the participants are presented in Table 1.

**Table 1:** List of demographic characteristics of the participants

No	Theme	Categories	N (number)
1	Gender	Female	30
		Male	15
2	Age (year)	25–30	6
		31–40	14
		41–50	20
		51 and above	5
3	Race	Malay	25
		Chinese	15
		Indian	5
4	Relationship with the cancer patient	Child	20
		Spouse	15
		Parent	10
5	Experience of caregiving	3 months to a year	25
		1 to 5 years	15
		More than 5 years	5

### Data analysis

A professional translator who speaks both Bahasa Malaysia and English was used to check the translated interview transcripts. A thematic analysis was used to identify codes and themes in the data collected. The analysis steps include data familiarization, initial code generation, review, and theme identification. The data was reread several times to identify the codes. SS and NAMH followed the same steps, and their answers were compared and discussed in order to reach a mutual agreement. The researchers’ backgrounds in health and qualitative studies helped to ensure accuracy in the data analysis.

### Trustworthiness of the data

Trustworthiness in qualitative studies is vital. There are four types of trustworthiness in qualitative studies that include credibility, transferability, dependability, and confirmability. For this study, credibility was established through a rapport with the participants. In the interview, they were told that honesty was very important, not just finding the “correct” answers. A rigorous discussion of the entire study process and results was conducted with both researchers in order to establish credibility. Transferability was gained by understanding that this current study did not represent the whole cancer patient population. However, it provides a deeper understanding of cancer patients in this study. Finally, to establish confirmability, bracketing was conducted which entails excluding one’s own perceptions and experience throughout the research activity. The researchers also reviewed relevant literature to remain objective throughout the research process and only considered the participants’ experience without putting their own meaning to these experiences.

## Results

During the analysis, five main themes emerged from the responses. The themes are guilt and blame, guilt due to lack of self-capacity, guilt for neglecting others, no guilt at all, and discussion on guilt and blame as a caregiver.

Theme 1: Guilt and self-blame.

Some participants reported blaming patients for their illness. Since caring for patients requires full-time commitment, some people find themselves sacrificing the time that they have to take care of them, which ultimately makes caring for patients a difficult, thankless job. However, some participants reported feeling guilty after blaming others. A 26-year-old participant who is taking care of her sick mother shared,

Sometimes I feel guilty. I know my mom is struggling with her illness. She is weak physically and mentally. She can easily get angry. I feel guilty when I try to blame her for the illness. If she took care of her health, she would not get the disease and she could maintain her health. As a result, I must sacrifice some of the time I wish I could spend with my friends like the rest of the world. I must look after her.

Similar to a 40-year-old mother who is taking care of her sick son, in her opinion, the illness is caused by her ignorance about not taking care of her son. She feels guilty and self-blames herself for her son’s actions. She said,

Sometimes I feel like I am a terrible mother. I read that an unhealthy diet, unhealthy lifestyles, and stress are factors that lead to cancer. Somehow, maybe he got it because I did not take care of him.

A 46-year-old husband feels guilty for blaming God for his wife’s illness and suffering. However, after a while, he began to make sense and feel guilty about the blame. He compares the things he has with the test he is taking now. He said,

There are times that I feel guilty when I blame God for giving her this disease. There are people who deserve this pain more than she does. However, I think about it and I feel terrible. God has given us so many things in this world including support, money, energy, and love. How could I blame Him for this test?

This 44-year-old wife expressed guilt over not being able to maintain her own health due to her responsibilities as a wife, mother, and daughter. She reported feeling mentally exhausted, but she had no choice but to act strong as everyone was depending on her. She shared,

This is weird, I guess. I have to do lots of things including providing care for my husband, kids, and parents. I have to work, handle daily chores. Sometimes I forget about myself. I feel guilt about my body. I am not taking care of my body at all. I feel so exhausted most of the time. But I must act like a superwoman. Everyone depends on me.

In a 42-year-old wife’s report, she felt guilty for feeling irritable while caring for her husband. Because of her irritability, she is easily angry and doubts God’s responsibility for the job. She reported,

Sometimes I feel guilty about being grumpy. I am tired and caring for a patient is never easy. I can easily get angry. I ask God, why? Why do I have to experience all this?

Theme 2: Guilt due to lack of self-capacity.

A 55-year-old woman expressed her guilt over not being able to assist her husband because of her own limitations. She said,

It is very painful to see him suffering. I just do not know what to do. I feel guilty for not being able to remove his pain. I am not a medical doctor. I wish I could do something.

A 27-year-old son reported feeling guilty for not being able to provide care for his sick mother. He felt guilty for letting his sister take care of their mother alone. Nevertheless, he also took responsibility to take care of his mother while letting his sister have her own time. He shared,

I wish I could spend more time with her, but I have to work. I am glad that my sister is taking care of her, but I feel guilty for letting her take care of our mother. She quit her job to take care of my mother. When I am not working, we take care of our mother together. It is important so my sister can also spend time doing what she likes.

Theme 3: Guilt for neglecting others.

Some participants reported feeling guilty of taking more time to provide care to the patient and neglecting some people around them. A 30-year-old mother shared her guilt over neglecting other children that she has. She claimed,

I feel guilty for my other kids and husband. I focus too much on my ill child. It is not that I do not care but providing care to an ill person needs more time and effort. My husband supports me, and we both help each other.

Theme 4: No guilt at all.

According to some participants, they did not feel guilty at all because they believed that the experience was a chance to be rewarded by God. A 41-year-old daughter described the experience as a chance for her to repay her parents’ sacrifices. She also reported feeling grateful for the support of her husband and kids. She said,

I do not feel guilty at all. Whatever is happening is God's will. I believe it is a chance for me to repay the kindness of my parents that took care of me and my siblings when we were small. It is a chance for me to gain heaven. Why should I blame God and feel guilty? I have done my best. I am so grateful that people around me are so supportive. Even my husband and kids help me.

Several family caregivers of patients with cancer reported understanding their roles as family caregivers of patients with cancer. Despite their importance to cancer patients, they believe they need to understand their limitations as human beings and the power of God. A 46-year-old husband reported,

Human beings are weak. We do our best, but God knows better. I know my mom knows that I am trying my best to provide assistance and support to her. Sometimes, I tell her how much I love her and though I cannot fulfil her needs 100%, I am trying and will always try.

Theme 5: Discussion on guilt and blame as a caregiver.

Some family caregivers of cancer patients have reported that the caregiving process can be challenging, leading to feelings of guilt and blame. However, guilt and blame are rarely discussed with others. According to a 46-year-old husband,

It is prohibited to discuss blame or guilt. As an Asian family, we live together for many reasons. Someone might accidently inform others and everyone will be informed about it, including the patient. There may be conflict in the family as a result of the situation. We do not want the patient to know about our struggle. If the patient knows about it, she must be very frustrated. Avoid it.

According to a 41-year-old daughter, discussing guilt and blaming is prohibited. She further explained that as a true believer, she must accept whatever God gives her without questioning it. She said,

I do not think guilt discussion is appropriate. Those who believe in God must accept what God has destined for them without asking why. Thus, I avoid discussing my feelings publicly or with close friends and family members. Cancer patients are tough, but I am my test. Do not talk about your feelings or challenges, just accept them.

## Discussion

This study aimed to explore the dynamics of guilt feelings in family caregivers of patients with cancer and how guilt influences their caregiving experience. There were several themes identified such as guilt and blame, guilt due to lack of self-capacity, guilt for neglecting others, and no guilt at all. Family caregivers of patients with cancer often feel guilt, but rarely discuss it in specific culture within society.

It has been suggested that guilt plays a significant role in the onset and maintenance of depression and anxiety, suggesting a close connection between guilt-related feelings and distress [24]. According to the Cancer Family Caregiving Experience Model [10], guilt is a significant aspect that can be influenced by carers’ stress processes, environmental circumstances, and cancer trajectory. The family caregiver also experiences anxiety and depression that are influenced by adults with cancer-related and family caregiver–related factors. The illness experienced by the cancer patients heightened stress in family caregivers with cancer patients as some reported of feel hopeless by seeing the patients suffer and unable to help them. At the same time, the family caregivers with cancer patients also experience challenges as they have to cope with their daily activities but still provide the care to the cancer patients. The inability to cope with the challenges can lead to anxiety and depression among themselves.

Additionally, providing care to the cancer patients is taking time and demanding prolonged time and energy. Therefore, the family caregivers with cancer patients have to change their lifestyles to adapt with the caring responsibilities. There is evidence that family caregivers of cancer patients who do not receive adequate time away from caregiving responsibilities experience a greater sense of burden [35]. The burden of care can be upsetting and leads to shame, embarrassment, guilt, and self-blame [33]. Therefore, the family caregivers of patients with cancer in this current study reported to experience blaming to other people and God as they are burnout and overwhelms of their roles.

In addition, family caregivers of patients with cancer reported feeling guilty about not being able to provide enough and quality care for patients. Additionally, the study indicates that the family caregivers of patients with cancer feel guilty for not being able to cope with the pain of the patients. There is a common occurrence when family caregivers of patients with cancer assume they should provide more care for cancer patients. Family caregivers of patients with cancer guilt are described as a sense of inadequacy in one’s perspective of the care offered to the care recipient [43]. Family caregivers of patients with cancer who feel less competent in their role as family caregivers of patients with cancer may become more stressed [35]. As a result, some family caregivers of patients with cancer in this study reported experiencing burnout. Family caregivers of patients with cancer often suffer from burnout [35].

Since family caregivers of patients with cancer experience burnout, they might experience self-blaming and guilt when they think that they failed to provide enough support to patients. Guilt has also been suggested as a factor potentially contributing to depression and distress in non-dementia caregiving family [35]. Family caregivers of patients with cancer feel motivated to improve the caregiving experience. If family caregivers of patients with cancer do not have positive appraisals and efficient coping strategies for the individual, the situation becomes much more severe [10]. The family caregivers of patients with cancer will feel stressed, especially when they do not have a good coping mechanism. Family caregivers of patients with cancer are too focused on providing care for patients.

Furthermore, this study also reported that family caregivers of patients with cancer feel guilty about other people such as other healthy kids because they are so busy caring for cancer patients. Family members with children with chronic illnesses often face this issue, which may lead to a lack of care for other siblings. A recent study found that kids in families with chronic illness reported that they were given differential parental treatment, extra caregiving or domestic responsibilities, and insufficient parent–child communication, as well as being affected by the perceived distress of their parents. Therefore, the family and usually the parents may feel guilty for not being able to provide support equally to all of their children due to their roles as family caregivers of patients with cancer.

There are also family caregivers of patients with cancer who do not feel guilty at all. Malaysians are religious people [40]. A study on parental grief in Malaysia reported that bereaved parents believed that whatever happens in their lives has been fated by God. Therefore, they need to accept the situation fully [26]. According to the findings of this study, family caregivers of patients with cancer believe that they have provided the most effective care to patients. Everything else is because of God’s will. In other words, the caring experience has been reported to generate positive growth and a feeling of appreciation for others. The lack of a sense of guilt has been reported to boost personal confidence and self-efficacy [14]. Thus, it shows that treating cancer patients is not always stressful. The caregiving experience helps family caregivers of patients with cancer to find strength and develop themselves in constructive ways.

The topic of guilt among family caregivers of cancer patients is rarely discussed in certain cultures, such as the Asian culture. Asian cultures also refrain from negative feelings or complaints [36]. Discussion about guilt can expose the whole family to rumors that affect family harmony. Therefore, refraining from discussing sensitive issues is vital. Asian families maintain their self-esteem and harmony of the family group [36]. Additionally, a discussion on guilt can also be viewed as a lack of religious belief that may affect faith in one’s religion. Additionally, a study on parental grief in Malaysia described that bereaved parents were more concerned with their religious beliefs than their emotional needs [26]. Family caregivers of cancer patients in this current study perceived religion as more critical to their own emotional needs to discuss their guilt and blame feelings.

## Study limitations

There were a relatively small number of participants in this qualitative study, making it impossible to generalize the findings to cancer patients’ families. In addition, this study employed data collection using social media which only involved those with access to social media.

## Data Availability

Not applicable.
